# SNAREs Regulate Vesicle Trafficking During Root Growth and Development

**DOI:** 10.3389/fpls.2022.853251

**Published:** 2022-03-14

**Authors:** Changxin Luo, Yumei Shi, Yun Xiang

**Affiliations:** MOE Key Laboratory of Cell Activities and Stress Adaptations, School of Life Sciences, Lanzhou University, Lanzhou, China

**Keywords:** Arabidopsis, vesicle trafficking, SNAREs, membrane fusion, root

## Abstract

SNARE (soluble N-ethylmaleimide-sensitive factor attachment protein receptor) proteins assemble to drive the final membrane fusion step of membrane trafficking. Thus, SNAREs are essential for membrane fusion and vesicular trafficking, which are fundamental mechanisms for maintaining cellular homeostasis. In plants, SNAREs have been demonstrated to be located in different subcellular compartments and involved in a variety of fundamental processes, such as cytokinesis, cytoskeleton organization, symbiosis, and biotic and abiotic stress responses. In addition, SNAREs can also contribute to the normal growth and development of Arabidopsis. Here, we review recent progress in understanding the biological functions and signaling network of SNAREs in vesicle trafficking and the regulation of root growth and development in Arabidopsis.

## Introduction

Plant cells contain multiple membrane-bound organelles, each of which contains a unique set of lipids and proteins that play different functions within and between cells. Membrane transport pathways connect these organelles, which are important for maintaining cell function and responding to various environmental stimuli. Vesicle trafficking involves vesicle formation, vesicle translocation, vesicle binding, and fusion of vesicles with target compartments. Vesicle formation is the process of vesicle bud formation from the donor compartment for cargo packaging and is mediated by the coat protein complex I (COPI), COPII and clathrin, and the small GTPases secretion-associated RAS super family 1 (Sar1) and ADP-ribosylation factor 1 (Arf1; Bremser et al., 1999; Bonifacino and Lippincott-Schwartz, 2003; Bonifacino and Glick, 2004). Vesicle translocation is mediated by motor proteins that propel vesicles along the cytoskeleton (Kamal and Goldstein, 2002). Tethering and Rab proteins regulate the docking of vesicles and receptor compartments (Grosshans et al., 2006; Verhage and Sørensen, 2008). Fusion is the final step of vesicle transport, mediated by the SNARE (soluble N-ethylmaleimide-sensitive factor attachment protein receptor) protein family (Hong, 2005; Chen et al., 2021).

SNAREs are a highly conserved superfamily of proteins that mediate vesicle transport between endosomes and the trafficking to the plasma membrane of all eukaryotic cells (Jahn and Scheller, 2006). Some SNARE proteins in plants have been found in various intracellular trafficking pathways and are involved in other physiological processes (Uemura et al., 2004), such as cell cytokinesis (Lauber et al., 1997; Collins et al., 2003; El Kasmi et al., 2013;Park et al., 2018), defense responses (Kwon et al., 2008, 2020; Gu et al., 2017; Yun and Kwon, 2017; Kim et al., 2019; Wang et al., 2020), shoot and root gravitropism (Kato et al., 2002; Morita et al., 2002; Yano et al., 2003; Gu et al., 2021), osmotic stress tolerance (Zhu et al., 2002), salt stress responses (Salinas-Cornejo et al., 2021; Sun et al., 2021), and ion channel regulation (Honsbein et al., 2009; Zhang et al., 2015a, 2020; Waghmare et al., 2018).

Arabidopsis primary root growth and development are regulated by elongation and cell division (Beemster and Baskin, 1998; Beemster et al., 2002; Lavrekha et al., 2017; Vyplelová et al., 2017). SNAREs regulate vesicle trafficking and are necessary for root growth, for example, the vesicle trafficking of SNAREs is important for the transport of the auxin transporter PINFORMED (PIN) in roots (Shirakawa et al., 2010; Gu et al., 2021; Zhang et al., 2021). Cytokinesis, the final step of cell division, physically separates the two daughter cells (Frémont and Echard, 2018), and SNARE complexes of different components together mediate the membrane fusion of Arabidopsis cytokinesis (Zhang et al., 2011a; El Kasmi et al., 2013; Park et al., 2018). This review begins with a description of the SNARE protein and SNARE-related vesicle transport pathways. Then, we predominantly focus on recent insights into the regulation of Arabidopsis root growth and how SNAREs participate in cytokinesis.

## Snare Proteins

The SNARE protein was first identified in the late 1980s and was quickly identified as a key element involved in membrane fusion (Oyler et al., 1989; Söllner et al., 1993). SNARE proteins form a superfamily of small proteins, there are 64 members in Arabidopsis, 25 members in Saccharomyces cerevisiae, and 36 members in humans (Jahn and Scheller, 2006; Zhang et al., 2020). The SNARE domain contains a SNARE motif with 60–70 amino acids and it consists of seven repeats that form a coiled-coil structure. Through hetero-oligomer interactions, SNAREs mediate the fusion of membranes and intracellular vesicle-related transport processes, which occur in vesicles, the inner membrane system of organelles, and the plasma membrane (PM; Lipka et al., 2007).

The relationship between SNAREs and the lipid bilayer occurs through the C-terminal transmembrane (TM) domain. Although most SNAREs are inserted into the cell membrane through transmembrane motifs, some SNAREs, such as Qbc-SNARE synaptosomal-associated 25 (SNAP25) and R-SNARE YKT6, are associated with posttranslational lipids of the peripheral junction membrane. In addition to the SNARE domain and C-terminal TM domain, many SNAREs also contain N-terminal regulatory motifs, which together with a series of accessory peptides control the activity of SNARE proteins *in vivo* (Fasshauer et al., 1998; Hong, 2005; Sutter et al., 2006; Lipka et al., 2007).

**Figure 1:**
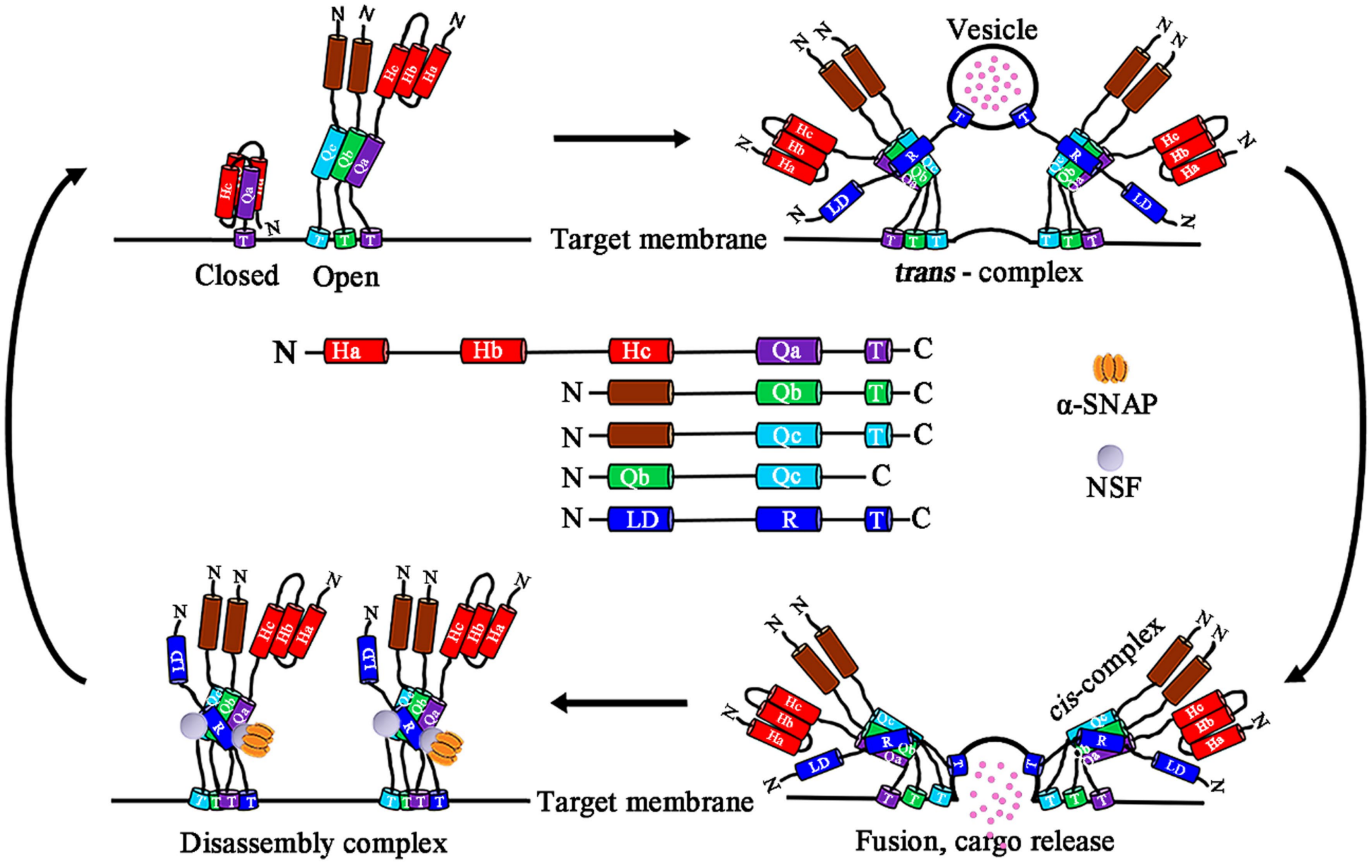
Schematic model domain structure of plant SNARE subfamilies and a model of SNARE complex vesicle fusion. Plant SNARE protein domains, including Qa, Qb, and Qc domains and regulatory N-terminal regions of Q-SNAREs, the R domain and the longin domain (LD) of R-SNAREs, and the C-terminal transmembrane helices (T), are present in most SNARE proteins. Qa-SNARE protein moving form a closed to open conformation that allows assembly with helices provided by membrane-associated Qb-, Qc-, and R-SNAREs. R-SNAREs reside in a vesicle. The assembly of a trans-SNARE complex is accompanied by an increase in the density of the core α-helical structure that transitions to the cis-complex and membrane fusion. Dissociation of the cis-complex requires the energy input of ATP hydrolysis and is achieved through the binding of α-SNAP and the NSF ATPase. Qa, Qb, Qc, and Q-SNARE three domains; R, R-SNARE domain; N, N-terminal; C, C-terminal; Ha, Hb, and Hc, the three helices of the Qa-SNARE N-terminal autoregulatory domain; LD, Longin domain; T, Transmembrane helices; α-SNAP, α-soluble NSF attachment protein; and NSF, n-ethylmaleimide-sensitive factor.

According to their subcellular localization, SNAREs are divided into two types: vesicle-associated (v-SNARE) and target membrane-associated (t-SNARE; Söllner et al., 1993; Jahn and Scheller, 2006). Depending on the glutamine (Q) or arginine (R) residue in the middle of the SNARE domain, it can be divided into Q- and R-SNAREs. In fact, t-SNAREs and v-SNAREs correspond to Q-SNAREs and R-SNAREs, respectively. R-SNAREs located on vesicles are usually called vesicle-associated membrane proteins (VAMPs). According to their sequence similarity, Q-SNAREs are divided into three subgroups: Qa-, Qb-, and Qc-SNAREs (Fasshauer et al., 1998; Jahn and Scheller, 2006; Lipka et al., 2007; Bock et al., 2011; Gu et al., 2020). Qa-SNAREs contain an autoregulation domain at the N-terminus, and this self-inhibitory domain is comprised of three helices, which are called Habc motifs in neuronal synapses. The Habc motif interacts with the SNARE domain in the same polypeptide. This intramolecular interaction is called the “close” conformation and prevents the assembly of Qa-SNARE with other SNAREs. Closed Qa-SNAREs unfold to form an active conformation (also called the “open”) and allow the formation of complexes. The Qa-Qb-Qc-cis SNARE complex and R-SNARE (v-SNARE) create a functional fusion of the trans-SNARE complex on the target membrane. After membrane fusion, SNARE complexes are transformed from a trans- to cis-configuration. Then, α-soluble NSF attachment protein (α-SNAP) mediates the breakdown of the SNARE complex by recruiting n-ethylmaleimide-sensitive factor (NSF) and activating its ATP enzyme activity, thereby releasing SNARE component units, and the cycle can then restart (Figure 1; Burgoyne and Morgan, 2007; Toonen and Verhage, 2007; Ryu et al., 2015; Zhao et al., 2015; Jun and Wickner, 2019; Chen et al., 2021; Song et al., 2021).

## Snares in Trafficking Pathways

**Figure 2:**
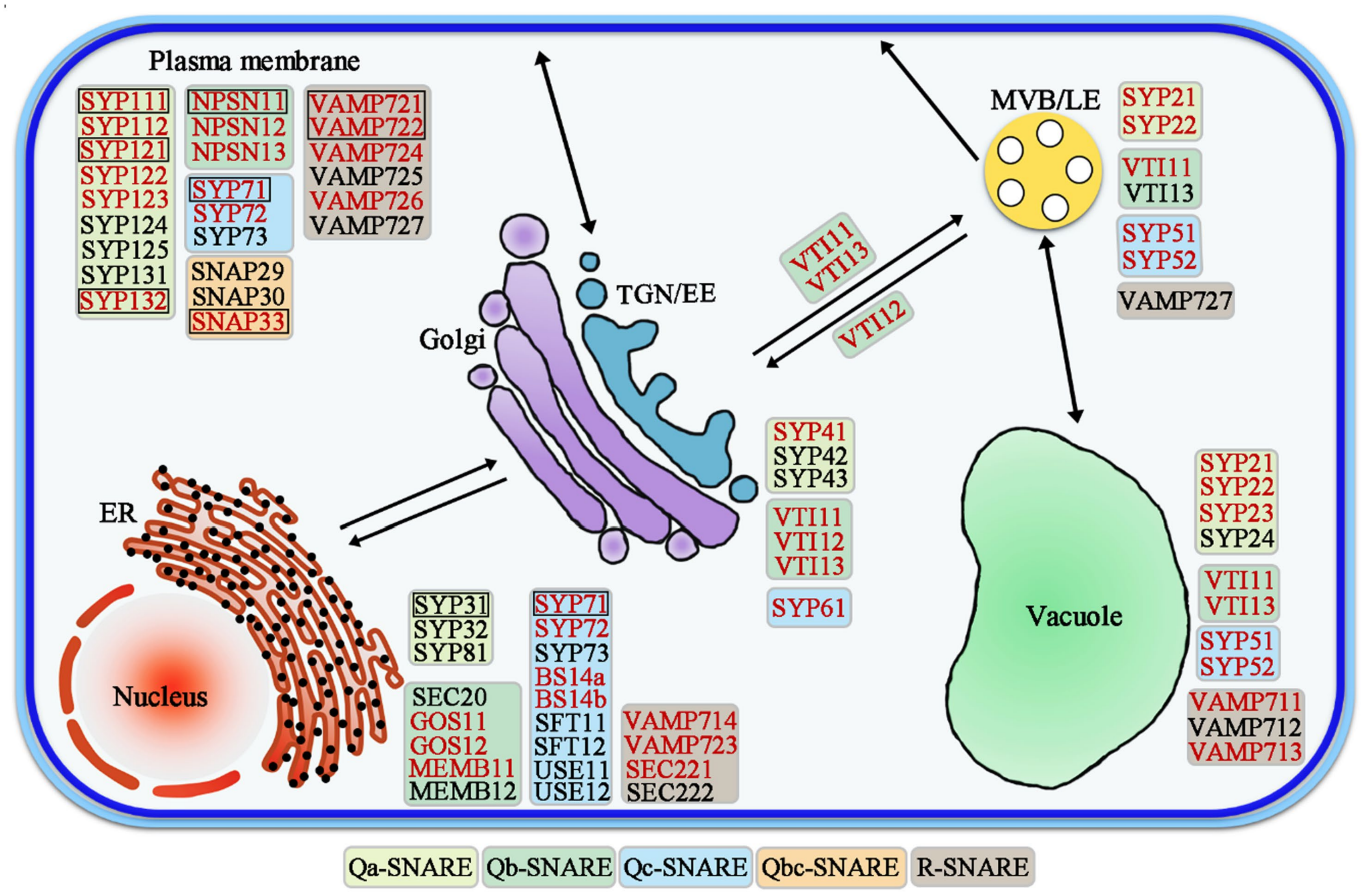
Schematic model of SNARE-mediated vesicle trafficking pathways in Arabidopsis. The genes highlighted in red are those that are highly expressed in roots. The black boxes mark the genes that are located on the cell plate. EE, Early endosome; LE, Late endosome; TGN, Trans-Golgi network; MVBs, Multivesicular bodies; and ER, Endoplasmic reticulum.

SNAREs are involved in different vesicle trafficking pathways (Figure 2). The majority of Arabidopsis SNAREs is located in a specific intracellular compartment, but some SNAREs have multiple patterns of localization in two or more organelles (Uemura et al., 2004; Sanderfoot, 2007; Bassham et al., 2008; Kim and Brandizzi, 2012). Various subcellular parts of plant cells form specific SNARE complexes that mediate various transport events (Kim and Brandizzi, 2012). SNAREs are key molecules involved in vesicle transport and membrane fusion, and they are also involved in different processes of the vesicle transport pathway, e.g., ER-Golgi anterograde/retrograde trafficking, trans-Golgi network (TGN) and post-Golgi trafficking, and the plasma membrane (Martinière and Moreau, 2020). SNAREs regulate transport in a complex membrane system, including endocytosis, secretory, and vacuolar trafficking steps in Arabidopsis (Sanderfoot, 2007; Saito and Ueda, 2009).

A total of 21 SNAREs are localized in the endoplasmic reticulum (ER) and Golgi (Figure 2; Table 1; Kim and Brandizzi, 2012). Qa-SNAREs syntaxin of plants 31 (SYP31) and SYP32 is located in the Golgi, and SYP31 is located at the formation cell plate in Arabidopsis (Sanderfoot et al., 2000; Rancour et al., 2002; Uemura et al., 2004). In pollen with syp31 syp32 double mutations, secretion of JIM7 (labeled highly methylesterified pectins)-positive vesicles from the Golgi/trans-Golgi network into the ectoplasm is blocked. Partial loss of the association of the endomembrane protein12 (EMP12) with the Golgi apparatus in syp31 syp32 double mutations pollen (Gao et al., 2012; Li et al., 2019; Rui et al., 2021). Furthermore, mCherry-HDEL trafficking between the ER and Golgi was disrupted, and it was mistargeted to vacuoles. SYP31 and SYP32 interact directly with conserved oligomeric Golgi 3 (COG3), which is a subunit of the COG complex and is responsible for its Golgi localization, indicating a role of SYP31/32 in intra-Golgi trafficking (Tan et al., 2016; Rui et al., 2021). Qc-SNARE BET12 localizes to both the Golgi and TGN, and it is involved in protein transport in the early secretory pathway. BET12 ectopic expression caused no inhibition in the ER-Golgi anterograde transport but caused intracellular accumulation of the antimicrobial protein PR1 (Pathogenesis-Related Gene 1). BET12 interacts with the Golgi-localized Qb-SNARE MEMB12, and MEMB12 overexpression accumulated PR1 in intracellular membranes. MEMB12 could be involved in retrograde protein trafficking from the Golgi back to the ER, and therefore, PR1 could be recycled to the ER instead of being secreted (Zhang et al., 2011b; Chung et al., 2018).

**Table 1:** Characteristics of SNAREs located in the ER/Golgi.

Type	Gene	Locus	Localization	Major phenotypes	Reference
Qa	SYP31	AT5G05760	Golgi	Syp31 mutant no noticeable phenotype.	Rui et al., 2021
	SYP32	AT3G24350	Golgi	The syp31 syp32 double mutant male gametophytic lethal.	Rui et al., 2021
	SYP81	AT1G51740	ER	Overexpression of AtSYP81 is shown to inhibit both retrograde and anterograde transport between the ER to Golgi in tobacco leaf protoplasts.	Bubeck et al., 2008 Martinière and Moreau, 2020
Qb	SEC20	AT3G24315	ER	Have not been characterized.	Li et al., 2013
	GOS11	AT1G15880	Golgi	?	Bassham et al., 2008
	GOS12	AT2G45200	Golgi	?	
	MEMB11	AT2G36900	cis-Golgi	Membrane trafficking at the ER-Golgi interface, act as a cis-Golgi recruiter of the GTPase Arf1.	Marais et al., 2015
	MEMB12	AT5G50440	Golgi	The memb12-1 has no obvious developmental defects, but shows increased resistance to Pst.	Zhang et al., 2011b Chung et al., 2018
Qc	USE11	AT1G54110		?	Bassham et al., 2008 Kim and Brandizzi, 2012
	USE12	AT3G55600		?	
	SYP71	AT3G09740	CP/PM/ E/ER	The syp71amiR and syp71 mutant has an abnormally severe phenotype, deformed cotyledons, and abnormal roots.	El Kasmi et al., 2013
	SYP72	AT3G45280	ER/PM	?	Moreau et al., 2007
	SYP73	AT3G61450	ER/PM	The primary root and elongation zone length of syp73 is shorter, and the fresh weight of the whole seedling is reduced.	Moreau et al., 2007 Cao et al., 2016
	BET11 (BS14a)	AT3G58170	ER/TGN/MVB	The bet11 single mutant has a shortened pollen tube and a germination rate of 63%.	Bolaños-Villegas et al., 2015 Delgadillo et al., 2020
	BET12 (BS14b)	At4G14455	ER/Golgi/TGN	The bet12 show reduced pollen tube length and the bet11 bet12 double mutants were more severe, overexpressing bet12 mutant exhibited short petioles and hypocotyls, insensitive to brassinolide (BL).	Zhu et al., 2014 Bolaños-Villegas et al., 2015 Chung et al., 2018 Delgadillo et al., 2020
	SFT11	AT4G14600	ER	?	Bassham et al., 2008
	SFT12	AT1G29060	Golgi	AtSFT12 OX are more resistant to salt and osmotic stresses and the atsft12 mutant is sensitive.	Tarte et al., 2015
R	VAMP714	AT5G22360	Golgi	The vamp714 loss of function and dominant negative and OX mutants exhibits a dwarf, excessive leaves and branches, shorter primary root and fewer lateral roots.	Uemura et al., 2004 Gu et al., 2021	
	VAMP723	AT2G33110	ER	?	Uemura et al., 2004	
	SEC221	AT1G11890	ER/Golgi/ Cytoplasm	AtSEC22 knockdown mutant, sec22-4 shows shorter primary roots, dwarf plants, sterility, epidermal cells were abnormal. Loss of SEC22, sec22-42 show Golgi fragmentation and pollen development was defective.	Chatre et al., 2005 El Kasmi et al., 2011 Guan et al., 2021	
	SEC222	AT5G52270		?	Bassham et al., 2008

There are 21 SNAREs localized in the TGN, endosomes, and vacuoles (Figure 2; Table 2; Kim and Brandizzi, 2012; Ito and Boutté, 2020). R-SNAREs vesicle-associated membrane protein 721 (VAMP721) and VAMP722 have been confirmed to be located in the PM; TGN/EE, VAMP721, and VAMP722 are involved in endocytosis of FM4-64 and the secretion and recycling of the PINFORMED 2 (PIN2) transporter in PM (Lipka et al., 2007; Zhang et al., 2011a, 2021; Uemura et al., 2019). Arabidopsis tomosyn protein (AtTMS) localizes to the trans-Golgi network, PM, and cytosol, where it can interact with several Qa-SNAREs through its C-terminal R-SNARE-like motifs. In some cases, overexpressed AtTMS binds to syntaxins and blocks secretion during pollen development. Transmission electron microscopy showed irregular membrane structure aggregation under the PM, but the Golgi stack looks normal (Larson, 2019; Li et al., 2019). These results suggest that the R-SNAREs VAMP721, VAMP722, and AtTMS mediate post-Golgi trafficking. The negatively dominant form of SYP22 (SYP22ND) is in the cytoplasm, while SYP22 is in the cytoplasm and vesicle-like compartments in tobacco leaves. The syp22 vamp727−/+ double mutants and syp22nd mutants are not sensitive to BRASSINOSTEROID (BR) treatment and BRI1 recycling to the PM is defective in syp22nd plants. VAMP727 and SYP22 interact with BRASSINOSTEROID INSENSITIVE (BRI1; Jones et al., 2014; Zhang et al., 2019b). SYP22 and VAMP727 are involved in BR signaling *via* regulation of BRI1 trafficking and they regulate plant defense by controlling the abundance of BRI1 on the PM (Zhu et al., 2019; Zhang et al., 2019a). The syp22 vamp727 double mutant contains several small vacuoles instead of the large vacuoles that occur in the wild type and vamp727, syp22 mutants. SYP22 and VAMP727 can form a complex for membrane fusion between the prevacuolar compartment (PVC) and vacuoles (Sanderfoot et al., 1999; Ebine et al., 2008).

**Table 2:** Characteristics located in the TGN/endosome, vacuole SNAREs.

Type	Gene	Locus	Localization	Major phenotypes	Reference
Qa	SYP21	AT5G16830	PVC/MVBs/Vacuole	The syp21 syp22 double mutant gametophyte lethality, female gametophyte lower viability.	Shirakawa et al., 2010 Touihri et al., 2011
	SYP22	AT5G46860	PVC/LE/ Vacuole	The syp22 mutant showed a semidwarf, poor leaf vein development and late flowering.	Ebine et al., 2008 Shirakawa et al., 2010 Ibrahim et al., 2020
	SYP23	AT4G17730	PVC/LE/Vacuole/Cytoplasm	Syp21amiR syp22 syp23 triple mutant growth and vein pattern defect.	Shirakawa et al., 2010 Ibrahim et al., 2020
	SYP24	AT1G32270	LE/Vacuole	?	Bassham et al., 2008
	SYP41	AT5G26980	TGN	The syp41 mutant has no apparent abnormalities. syp41 syp42 double mutants show a shorter root.	Sanderfoot et al., 2001 Uemura et al., 2012
	SYP42	AT4G02195	TGN ?	The syp42 mutant has a slightly short root. The syp42 syp43 has short roots, many lateral roots, semidwarfism, and early senescence.	Uemura et al., 2012
	SYP43	AT3G05710			
Qb	VTI11	AT5G39510	TGN/PVC/ Vacuole	Vti11 mutant has vacuole morphology defects and defects in shoot gravitropism.	Yano et al., 2003 Niihama et al., 2005 Zheng et al., 2014 Cabanillas et al., 2018
	VTI12	AT1G26670	PM/TGN/ PVC	The vti12 mutant has defects in the autophagy pathway and the vti11 vti12 double mutant is lethal.	Surpin et al., 2003 Niihama et al., 2005 Sanmartín et al., 2007
	VTI13	AT3G29100	Golgi /TGN /PVC/ Vacuole	Vti13 seedlings root hairs are shorter and exhibit branching as well as sensitivity to mannitol.	Uemura et al., 2004 Larson et al., 2014
	VTI14	AT5G39630	?	?	Kim and Brandizzi, 2012
Qc	SYP51	AT1G16240	TGN /Vacuole	?	De Benedictis et al., 2013 Barozzi et al., 2019
	SYP52	AT1G79590	TGN /Vacuole	?	
	SYP61	AT1G28490	TGN/E/PM	The syp61 mutant had a more branched root and was extremely sensitive to the inhibition of Na+, K+ and Li+; opening of the stomata was impaired.	Sanderfoot et al., 2001 Zhu et al., 2002 Rosquete and Drakakaki, 2018
R	YKT61	AT5G58060	Cytoplasm/punctate vesicles	The ykt61 mutant male and female gametophytes was lethal.	Uemura et al., 2004 Ma et al., 2021	
	YKT62	AT5G58180	?	?	Chen et al., 2005	
	VAMP711	AT4G32150	TGN/PVC/ Vacuole	The vamp711 mutant is sensitive to drought stress, stronger resistance to high pH, stomatal movement is impaired.	Uemura et al., 2005 Leshem et al., 2006 Xue et al., 2018, 2019	
	VAMP712	AT2G25340	TGN/Vacuole	The vamp711 vamp712 vamp714 triple mutant shows stronger resistance to high pH stress.	Xue et al., 2018, 2019	
	VAMP713	AT5G11150	TGN/Vacuole	?	Takemoto et al., 2018	
	VAMP727	AT3G54300	P M /E/ PVC/Vacuole	The vamp727 mutant displays no visibly abnormal phenotype.	Takemoto et al., 2018 Zhang et al., 2021	
	VAMP728	AT3G24890	?	?	Bassham et al., 2008

There are 22 SNAREs localized in the PM (Figure 2; Table 3; Kim and Brandizzi, 2012; Ruan et al., 2021). Syntaxin SYP121 is a plasma membrane Qa-SNARE and it consists of N, H, Q, and C four regions. The SYP121 sequence deletion shows that the C region contains the transmembrane domain and the H and Q regions contain the Habc and Qa-SNARE functional domains, interacting with plasma membrane intrinsic protein 2;7 (PIP2;7), which is involved in membrane fusion. SYP1s, except SYP112, and SYP121 orthologs interact with PIP2,7 (Hachez et al., 2014; Zhang et al., 2019a; Laloux et al., 2021). The proteins, PICALM1a and PICALM1b, which contain the ANTH domain are used as adapter proteins of the secretory vesicle-associated VAMP72 group clathrin-mediated endocytosis (CME). Retrieving VAMP721 from the PM requires PICALM1 and the loss of this function will result in the accumulation of VAMP721 in the PM (Fujimoto et al., 2020).

**Table 3:** Characteristics located in plasma membrane SNAREs.

Type		Gene	Locus	Localization	Major phenotypes	Reference
Qa	SYP111	AT1G08560	CP/TGN/ MVB	Short roots, deformed cotyledons strong cytokinesis defects during embryogenesis.	Lukowitz et al., 1996 El Kasmi et al., 2013 Park et al., 2018
	SYP112	AT2G18260	PM	?	Bassham et al., 2008
	SYP121	AT3G11820	PM	In low humidity and strong light, the syp121 mutant has a low stomatal conductivity, inhibited vegetative growth. Disease resistance weakened.	Collins et al., 2003 Meyer et al., 2009 Honsbein et al., 2009 Eisenach et al., 2012 Laloux et al., 2021
	SYP122	AT3G52400	PM	The syp121 syp122 double mutant is severely dwarfed and partially necrotic and several defense pathways are active. Regulates the channel activity to promote K+ uptake.	Assaad et al., 2004 Schultz-Larsen et al., 2018 Waghmare et al., 2018 Zhang et al., 2019a
	SYP123	AT4G03330	PM	The syp123 causes severe defects in root hair elongation.	Ichikawa et al., 2014
	SYP124	AT1G61290	PM	Involved in the growth of the pollen tube tip.	Silva et al., 2010 Slane et al., 2017
	SYP125	AT1G11250	PM	Syp124 syp125 double mutant pollen tubes displayed no obvious defects.	Slane et al., 2017
	SYP131	AT3G03800	PM	The syp131 single mutant is normal, the triple mutant syp124 syp125 syp131 exhibits a specific and severe male gametophytic defect.	Slane et al., 2017
	SYP132	AT5G08080	PM/CP	The syp132amiR had significantly shorter root hair, syp132 mutant cytokinesis defects.	Kalde et al., 2007 Ichikawa et al., 2014 Park et al., 2018
Qb	NPSN11	AT2G35190	CP/TGN/ PM	The npsn11-1 plants showed no obvious phenotypes.	Zheng et al., 2002 El Kasmi et al., 2013
	NPSN12	AT1G48240	TGN/PM	The npsn12 mutant plants showed significantly increased leaf area and biomass in UV stress.	Piofczyk et al., 2015
	NPSN13	AT3G17440	TGN/PM	Have not been characterized	Bassham et al., 2008
Qbc	SNAP29	AT5G07880	PM	?	Kim and Brandizzi, 2012 Won and Kim, 2020
	SNAP30	AT1G13890	PM	?	
	SNAP33	AT5G61210	CP/PM/E	The snap33 mutant showed severe cotyledon necrosis and a fatal phenotype for seedlings.	Heese et al., 2001 El Kasmi et al., 2013 Won and Kim, 2020
R	VAMP721	AT1G04750	PM/TGN/EE/CP	The vamp721 or vamp722 single mutants display no obvious phenotypic, the vamp721 vamp722 double mutant seedlings have severely defective roots, hypocotyls and cotyledons, weakened resistance to extracellular pathogens.	Zhang et al., 2011a,^139,57^ Kwon et al., 2020 Kim et al., 2021	
	VAMP722	AT2G33120	PM/TGN/ EE/CP			
	VAMP724	AT4G15780	TGN/PM	?	Uemura et al., 2004 Sanderfoot, 2007 Kim and Brandizzi, 2012	
	VAMP725	AT2G32670	TGN/PM	?		
	VAMP726	AT1G04760	TGN/PM	?		
	AtTMS (TYN11)	AT5G05570	TGN/PM	The Attms mutants by CRISPR/Cas9- mediated gene editing have no obvious phenotype, AtTMS-OE affects the microspore stage of pollen development.	Li et al., 2019	
	TYN12	AT4G35560		?	Sanderfoot, 2007

## Snares Regulate Root Growth and Development

### SNAREs Affecting Cell Elongation/Cell Growth

In plants, root systems are the underground organs that develop from the root apical meristem initiated during embryogenesis, and they respond to a variety of environmental obstacles and stimuli. Not only do the roots provide structural support for the plant’s aboveground parts, but they also absorb nutrients and water, both of which are required for plant growth (Motte et al., 2019). Thus, root growth and development are critical to overall plant survival. Cell elongation and cell division are used to promote root tip growth (De Smet et al., 2015). Intracellular vesicle transport from the ER to the Golgi apparatus, plasma membrane, vacuole or lysosome, and endocytosis plays important physiological functions in regulating plant development. Many proteins, such as ADP-ribosylation factor (ARF), ARF-guanine exchange factors (ARF-GEFs), ARF-GTPase-activating proteins (ARF-GAPs), Rab GTPase, and Rho-related GTPase of plants (ROPs), or their regulators, such as phospholipids, sterol kinases, and phosphatase, regulate root growth through vesicular trafficking (Yao and Xue, 2011). SNAREs play an important role in different pathways of vesicular trafficking and fusion with specific organelles to regulate root growth.

#### ER and Golgi SNAREs in the Root

Eleven of the ER/Golgi pathway SNAREs are highly expressed in roots, including three Qb-SNAREs: GOS11, GOS12, and MEMB11; five Qc-SNAREs: SYP71, SYP72, SYP73, BS14a, and BS14b; and three R-SNAREs: VAMP714, VAMP723, and SEC221. These SNAREs are necessary for root growth and development of Arabidopsis (Table 1; Figure 2; Uemura et al., 2004; Lipka et al., 2007). After 3 days of germination, syp73 had a shorter primary root, a significantly reduced elongation zone, and a lower fresh weight of the whole seedling. Overexpression of SYP73 causes rearrangement of the ER over actin. In Arabidopsis, SYP73 anchors the ER to the actin cytoskeleton to maintain the integrity and fluidity of the ER (Moreau et al., 2007; Cao et al., 2016). R-SNARE SEC22 is located in the ER, Golgi apparatus, and cytoplasm (Guan et al., 2021). Sec22-4 shows shorter primary roots, dwarf plants, sterility, and abnormal epidermal cells. AtSEC22 plays an important role in membrane transport and cytoskeletal dynamics during plant development (Chatre et al., 2005; El Kasmi et al., 2011; de Marcos Lousa et al., 2016; Guan et al., 2021). The vamp714 transfer DNA (T-DNA) insertional mutants grown in soil develop a shorter primary root and fewer lateral roots, have abnormal gravity responses, and disordered root cell arrangements. In addition, VAMP714 overexpressing lines and dominant-negative mutants also show a reduced seedling root system. The Arabidopsis R-SNARE VAMP714 protein colocalized in the ER, Golgi vesicles, and PIN proteins on the plasma membrane. The auxin distribution pattern in the root tip of the vamp714 mutant is aberrant, and the content is low (Gu et al., 2021). VAMP714 is required for PIN1 and PIN2 recycling. The actin depolymerizing agent latrunculin B (Lat B) caused the accumulation of VAMP714 vesicles in the cell. VAMP714 is part of the extracellular vesicle transport pathway of the ER/Golgi and the actin-dependent endocytic circulation pathway, which together regulate the abundance of PIN proteins in the plasma membrane (Geldner et al., 2001; Gu et al., 2021). This suggests that VAMP714 regulates the endocytic circulation pathway of vesicle transport involved with the PIN proteins and that the circulation of endosomes is essential for root growth and development.

#### TGN/Endosome and Vacuolar SNAREs in the Root

Twelve of the TGN/endosome, vacuolar pathway SNAREs are highly expressed in roots: four Qa-SNAREs: SYP21, SYP22, SYP23, and SYP41; three Qb-SNAREs: VTI11, VTI12, and VTI13; three Qc-SNAREs: SYP51, SYP52, and SYP61; and two R-SNAREs: VAMP711 and VAMP713 (Table 2; Figure 2; Uemura et al., 2004; Lipka et al., 2007). The Qa-SNARE SYP4 group (SYP41, SYP42, and SYP43) is all located in the same TGN compartment and some function redundantly (Uemura et al., 2012). The root length of the syp42 mutant is slightly shorter than that of the wild type, but there is no obvious abnormality in the syp41 and syp43 mutants. The syp42 syp43 double mutant has short roots, a large number of lateral roots, semidwarfism, early senescence, the transport of defective PIN2-GFP to the vacuole, and visible accumulation of secGFP (a signal peptide added to a variant of GFP; Uemura et al., 2012, 2019). This suggests that SYP4s regulate multiple transport pathways in plants that are involved in root growth and extracellular disease resistance. The Qb-SNARE VTI13 localizes to the Golgi, TGN/EE, and vacuole membrane in root hair cells, the vti13 mutant has short and bifurcated root hairs, and osmotic stress can exacerbate this phenotype. VTI13 is involved not only in the transport of cargo to vacuoles but also in cell wall organization and has a role in root hair growth (Larson et al., 2014). Vacuolar SNARE VTI11 is required for auxin-dependent morphogenesis of vacuoles, and its loss of function makes cells significantly insensitive to the growth inhibitory effect of auxin (Löfke et al., 2015). Thus, SNARE-dependent vacuolar morphogenesis allows auxin to limit cell expansion and promote the growth rate of root organs. In Arabidopsis, R-SNARE VAMP7C: VAMP711-VAMP714 forms a complex that promotes the fusion of vesicles and tonoplasts (Uemura et al., 2004; Leshem et al., 2006). The vamp711 vamp712 vamp714 triple mutant showed greater resistance to high pH stress than the vamp711 mutant. However, the vamp711 mutant is also more resistant to high pH than the wild type. A luciferase complementation (LUC) assay showed that VAMP7C interacted with PM H^+^-ATPase 2 (AHA2) and was involved in the regulation of PM H^+^-ATPase activity (Xue et al., 2019). The tonoplast localized SNARE (VAMP711-YFP, SYP21-YFP, and SYP22-GFP) fluorescence intensity increases with increasing auxin biosynthesis or the administration of exogenous auxin.

#### PM-Associated SNAREs in the Root

Thirteen of the PM-associated SNAREs are highly expressed in roots: five Qa-SNAREs: SYP111, SYP112, SYP121, SYP122, and SYP132; three Qb-SNAREs: NPSN11, NPSN12, and NPSN13; one Qb + Qc-SNARE: SNAP33; and four R-SNAREs: VAMP721, VAMP722, VAMP724, and VAMP726 (Table 3; Figure 2; Uemura et al., 2004; Lipka et al., 2007). Double homozygous vamp721 vamp722 mutant seedlings stopped growing after 2 days of germination and produced extremely thick roots, hypocotyls, and cotyledons; the seedlings died after 10 days. In addition, compared with wild-type seedlings, the roots of the vamp721 vamp722 mutant showed disordered root tips, including abnormal meristematic cells and root caps (Zhang et al., 2011a). The vamp721 vamp722 mutant shows aberrant localization of PINs and AUX1 and an enlarged TGN structure at the root (Zhang et al., 2021). The Arabidopsis R-SNAREs VAMP721 and VAMP722 have been found in the PM, TGN/EE, and cell plate (Lipka et al., 2007; Zhang et al., 2011a; Uemura et al., 2019). VAMP721 and VAMP722 are involved in endocytosis of FM4-64 and the secretion and recycling of the PIN2 transporter in PM but not in vacuoles. The R-SNAREs VAMP721 and VAMP722 play important roles in mediating the post-Golgi trafficking of auxin transporters and seedling growth (Zhang et al., 2021). The small GTPase RABA2a recruits the ternary complex VAMP721/722-SYP121-SNAP33 and interacts with it to achieve membrane fusion. The plant-specific RABA2a-SNARE pathway is essential for maintaining potassium ion homeostasis (Pang et al., 2021). This suggests that the R-SNARE VAMP721/722 plays important roles in mediating vesicle trafficking to maintain Arabidopsis root growth. The Qa-SNARE SYP132 is primarily localized in the PM (Uemura et al., 2004; Xia et al., 2019). The root length of SYP132-overexpressing seedlings was shorter than that of the wild type. Auxin regulates SYP132 in root growth and the geotropic response. The expression of SYP132 is tightly regulated by auxin, and increased expression of SYP132 reduces the content of H^+^-ATPase protein on the plasma membrane and is involved in the endocytosis of plasma membrane H^+^-ATPase proteins, reducing their density and activity on the PM (Xia et al., 2019, 2020). The loss of function of SYP123 and SYP132 leads to serious defects in root hair elongation. SYP123, rather than SYP132, is located at the root hair tip region in an f-actin-dependent manner by circulating between brefeldin A (BFA) sensitive endosomes and the PM at the expanded root hair tip (Ichikawa et al., 2014). Cumulatively, SYP123 and SYP132 work together to mediate membrane transport at the root hair tip and to promote root hair tip growth.

### SNARES Affecting Cytokinesis

Plant morphogenesis is regulated by cell division and expansion (Bednarek and Falbel, 2002). Cell division is a major biological process that has been extensively studied. Similar to other eukaryotic cells, plant cells form mitotic spindles to divide replicated chromosomes (sun et al., 2018). Mitosis of plant cells is a dynamic process controlled by the rearrangement of microtubules that gradually transition into distinct arrangements of microtubules during mitosis and cytokinesis. Before cell division, cortical microtubules gradually narrow to form the preprophase band (PPB), which determines the level of cell division (Rasmussen et al., 2011). After the nuclear envelope ruptures and the mitotic process begins, microtubules form the mitotic spindle, which is responsible for dividing chromosomes into daughter cells during mitosis (Vos et al., 2004; Marcus et al., 2005; Azimzadeh et al., 2008). The final stage of cell division is cytokinesis, in which the bipolar spindle turns into the phragmoplast and acts as a guide to cell plate assembly and the subsequent formation of a new cell wall (Lee and Liu, 2013), which divides the cytoplasm and organelles and completes the formation of membrane barriers between the daughter cells, separating them (Bednarek and Falbel, 2002; Segui-Simarro et al., 2004; Jürgens et al., 2015). In Arabidopsis, the SNARE protein can promote the formation of cell plates (Figure 2; Lukowitz et al., 1996). The Qa-SNAREs SYP132 and KNOLLE form two SNARE complexes to regulate cytokinesis in Arabidopsis (El Kasmi et al., 2013; Park et al., 2018).

A cytokinesis-specific syntaxin KNOLLE (SYP111) is produced in the late G2/M phase, and it quickly reverses at the end of cytokinesis (Lauber et al., 1997; Völker et al., 2001; Reichardt et al., 2011). A newly synthesized KNOLLE protein is inserted into the ER membrane and travels along the secretory pathway to the plane of cell division *via* the Golgi stack and TGN, where it is endocytosed and directed to the vacuole *via* the multivesicular body (MVB) after cell plate formation (Reichardt et al., 2007; Karnahl et al., 2017). The cotyledons of all knolle seedlings are malformed, and the roots are short but clearly distinguishable. In weakly affected seedlings, the cotyledons are green and the roots have well-formed root hairs (Lukowitz et al., 1996). KNOLLE forms two SNARE complexes that are important in the cytokinesis process: a trimer complex comprised of KNOLLE and its companion Qbc-SNARE SNAP33 and R-SNARE VAMP721 or VAMP722 and a tetramer complex comprised of Qb-SNARE NOVEL PLANT SNARE 11 (NPSN11), Qc-SNARE SYP71, and R-SNARE VAMP721 or VAMP722 (El Kasmi et al., 2013; Karnahl et al., 2017; Park et al., 2018). KNOLLE, SNAP33, and NPSN11 are three Q-SNAREs found on the cell plate of dividing cells (Lauber et al., 1997; Heese et al., 2001; Zheng et al., 2002). Endogenous SYP71 accumulates on the cell plate, where it colocalizes with KNOLLE (El Kasmi et al., 2013). The snap33 mutant exhibits only minor cytokinesis defects during the seedling stage; however, severe cotyledon necrosis and seedling death occurred during seedling growth (Heese et al., 2001). There is no obvious cytokinesis defect in the npsn11 mutant, and homozygous plants are viable (Zheng et al., 2002). The roots of double mutant npsn11 syp71 seedlings are short, the root hair and hypocotyl grow well, and they may not have a cell division defect phenotype. The roots of snap33 syp71 double mutants are extremely short and exhibit a major cytokinetic defect, with dividing cells exhibiting abnormal morphology similar to that of knolle mutant (El Kasmi et al., 2013; Park et al., 2018). The vamp721 vamp722 double mutants had a defective cotyledon, vein patterning and root growth, and disordered root epidermis, cortex, and stele cell layer patterns, which displayed abnormal cell files (Zhang et al., 2011a, 2015b). Vamp721 vamp722 mutant seedlings exhibit cell wall stubs and incomplete cytokinesis and they inhibit the secretion of plasma membrane proteins. During cytokinesis, VAMP721 and VAMP722 are found on the cell plate (Zhang et al., 2011a; El Kasmi et al., 2013; Park et al., 2018). Concanamycin A (ConcA) treatment slows the expansion of the cell plate labeled with GFP-KNOLLE, resulting in cell wall stubs, as does intracellular accumulation of GFP-VAMP721 and GFP-VAMP722, so inhibiting trafficking at the TGN affects cell plate formation (Huss et al., 2002; Reichardt et al., 2007; Zhang et al., 2011a). VAMP721 and VAMP722 activities are required for secretory trafficking from the TGN to the cell plate in dividing cells and the plasma membrane (Zhang et al., 2011a). This suggests that the SNARE complex, which contains two types of KNOLLE, is functionally redundant and mediates cytokinesis in Arabidopsis.

SYP132 is a nonspecialized Qa-SNARE derived from an alga-like ancestor, and the SNARE partners for SYP132 and KNOLLE are the same. They have no overlapping functions in secretion, and the cellularization process of nourishing the embryonic endosperm is caused by the double fertilization unique to flowering plants (Park et al., 2018). *In vitro* and *in vivo* interactions occur between SYP132 and VAMP721/722, but not VAMP723 (Yun et al., 2013; Ichikawa et al., 2014). Qa-SNARE SYP132 is necessary for cytokinesis. SYP132 T-DNA insertion in the promoter region produces mutant syp132T seedlings that have abnormally formed adventitious roots instead of a single primary root. The SYP132 artificial microRNA mutant syp132amiR displays a disorganized shoot meristem and the hallmarks of defective cytokinesis as well as a very short root (Enami et al., 2009; Reichardt et al., 2011; Park et al., 2018). The combination of syp132amiR and syp132T alleles creates a SYP132 mutant with an enhanced mutant phenotype (syp132tam). Syp132tam mutant embryos and seedlings have a mutated phenotype that is difficult to distinguish from that of knolle mutant embryos and seedlings. Cytokinesis defects in syp132tam embryos include enlarged multinucleated cells and occasionally enlarged nuclei, and syp132tam embryos show bands of unfused vesicles (Weijers and Offringa, 2003; Park et al., 2018). Membrane vesicles delivered to the cell division plane fuse to form a partitioning membrane and require the SM protein KEULE to interact with KNOLLE in Arabidopsis cytokinesis. KEULE has the paralog SEC1B, which strongly preferentially interacts with the ancient Qa-SNARE SYP132, which is involved in secretion and cytokinesis, and KEULE also interacts with SYP132 (Karnahl et al., 2018). These results show that SYP132 is necessary for embryonic secretion and plays a role in embryonic cytokinesis. KNOLLE is necessary not only for somatic cytokinesis but also for cellularization of the endosperm.

## Perspective

Recent studies have fully demonstrated that SNAREs are a multifunctional protein family with a wide range of biological functions in plants, and they not only participate in the normal growth and development of plants by mediating vesicle fusion, but they also regulate vesicle transport to response to the external environment by cooperating with other factors. Through signal transduction, the whole plant coordinates to adapt to environmental changes and continues to survive and reproduce. However, there is limited information in plant genetics research on the role of SNAREs. Some SNARE mutants do not show obvious phenotypes under normal conditions, which could be due to functional redundancy among related family members, or it could perform a specific function under a specific type of stress. This will provide an interesting perspective for future research into the functional diversity of different homologous genes in protein transport and plant stress tolerance. SNARE function requires sequential dynamic interactions between different SNAREs, and other proteins may be involved in SNARE function; for example, the Qa-SNARE KNOLLE interacts with Q-SNAREs SNAP33 and SYP71 to mediate membrane fusion in Arabidopsis cytokinesis. SM proteins and α-soluble NSF participate in SNARE complex assembly and disassembly (El Kasmi et al., 2013; Yoon and Munson, 2018). SNAREs and Rab GTPases have been shown to functionally promote vesicle fusion synergy and improve membrane fusion specificity and efficiency (Ohya et al., 2009; Ebine et al., 2011). SNAREs have been discovered for many years, and the mechanisms underlying SNARE-mediated membrane fusion are well understood. However, there are a few unanswered questions. Root development in Arabidopsis is a dynamic process involving complex interactions between transcriptional regulators and plant hormones. SNARE regulates auxin transport and contributes to root development, and what factors in addition to auxin are involved in Arabidopsis root growth? How is SNARE-mediated vesicular trafficking coordinated, thereby controlling root growth?

In the process of cytokinesis, KNOLLE and SYP132 have serious cytokinesis defects in the embryonic stage, but some of them can grow into seedlings, how is cell fate finely regulated in this process? What is the biological significance of two distinct SNARE complexes that mediate the same process of membrane fusion during cytokinesis? More SNARE complexes and more SNARE interaction factors remain to be discovered. With further research, it is expected that the molecular fine-regulation mechanism of SNARE-mediated vesicle transport will become clearer in the near future, thus providing a more comprehensive understanding of the important role of this protein family in plant growth and development.

## Author Contributions

CL, YS, and YX wrote the manuscript. All authors contributed to the article and approved the submitted version.

## Funding

This work was supported by the National Natural Science Foundation of China (31970195).

## Conflict of Interest

The authors declare that the research was conducted in the absence of any commercial or financial relationships that could be construed as a potential conflict of interest.

## Publisher’s Note

All claims expressed in this article are solely those of the authors and do not necessarily represent those of their affiliated organizations, or those of the publisher, the editors and the reviewers. Any product that may be evaluated in this article, or claim that may be made by its manufacturer, is not guaranteed or endorsed by the publisher.
